# The Activation of Mesenchymal Stem Cells by Glioblastoma Microvesicles Alters Their Exosomal Secretion of miR-100-5p, miR-9-5p and let-7d-5p

**DOI:** 10.3390/biomedicines10010112

**Published:** 2022-01-06

**Authors:** Delphine Garnier, Edward Ratcliffe, Joséphine Briand, Pierre-François Cartron, Lisa Oliver, François M. Vallette

**Affiliations:** 1 CRCINA INSERM U1232, CHU de Nantes, Université de Nantes, 44000 Nantes, France; edward.ratcliffe@inserm.fr (E.R.); briand@ohsu.edu (J.B.); pierre-francois.cartron@inserm.fr (P.-F.C.); lisa.oliver@univ-nantes.fr (L.O.); francois.vallette@inserm.fr (F.M.V.); 2 LaBCT, Institut de Cancérologie de L’Ouest, 44800 Saint Herblain, France; 3 Centre de Recherche des Cordeliers, Sorbonne Université, INSERM, Université de Paris, 75006 Paris, France

**Keywords:** tumor microenvironment, extracellular vesicles, glioblastoma, MSC activation, exosomal miRNAs, CAFs

## Abstract

Glioblastoma (GBM) is the most aggressive brain tumor, and despite initial response to chemo- and radio-therapy, the persistence of glioblastoma stem cells (GSCs) unfortunately always results in tumor recurrence. It is now largely admitted that tumor cells recruit normal cells, including mesenchymal stem cells (MSCs), and components of their environment, to participate in tumor progression, building up what is called the tumor microenvironment (TME). While growth factors and cytokines constitute essential messengers to pass on signals between tumor and TME, recent uncovering of extracellular vesicles (EVs), composed of microvesicles (MVs) and exosomes, opened new perspectives to define the modalities of this communication. In the GBM context particularly, we investigated what could be the nature of the EV exchange between GSCs and MSCs. We show that GSCs MVs can activate MSCs into cancer-associated fibroblasts (CAFs)-like cells, that subsequently increase their secretion of exosomes. Moreover, a significant decrease in anti-tumoral miR-100-5p, miR-9-5p and let-7d-5p was observed in these exosomes. This clearly suggests a miRNA-mediated GBM tumor promotion by MSCs exosomes, after their activation by GBM MVs.

## 1. Introduction

Glioblastoma (GBM) is classified as grade IV brain tumor by the World Health Organization (WHO), with only a 14-month median survival rate for patients [1]. Treatment includes maximal surgical resection, radiotherapy and Temozolomide chemotherapy [2], but unfortunately, the initial therapy response is systematically followed by tumor recurrence. This resistance to treatment is driven by a specific subpopulation, the Glioblastoma stem cells (GSCs), that can self-renew or differentiate and therefore generate tumor heterogeneity, while being responsible of tumor growth initiation and post-therapy recurrence [3].

GBM tumor fate is also under the influence of normal cells surrounding the tumor, the tumor microenvironment (TME), composed of stromal cells such as mesenchymal stem cells (MSCs), endothelial cells, immune cells in addition of extracellular matrix. MSCs are multipotent cells, defined by their capacity to either self-renew or differentiate into adipocytes, osteoblasts or chondrocytes [4]. Added to this, MSCs are characterized by the expression of CD73, CD90 and CD105, and the absence of hematopoietic markers (CD45, CD34, CD14, CD11b, CD79a, CD19 or class II histocompatibility complex antigens) [5]. In a cancer progression context, MSCs naturally display a tropism towards tumor cells, where their number inversely correlates to GBM patients overall survival, suggesting a tumor promoting role [6]. However their contribution is not completely clear yet [7].

Communication between GSCs tumor cells and MSCs from the TME has been largely studied, however the recent discovery of extracellular vesicles (EVs) brought to light a new way to convey signals between the tumor and TME to insure a bidirectional communication [8]. EVs are particles released by cells and delimited by a lipid bilayer [9] that can be divided into 2 main populations: exosomes, which are very small (50–150 nm) membrane-derived vesicles generated through the endocytic pathway and microvesicles (MVs)(100–1000 nm) that are generated by blebbing of the plasma membrane [10,11,12]. They carry proteins, DNA, RNA as well as metabolites that constitute critical messengers contributing to tumor growth, dissemination and drug resistance [13,14].

The participation of EVs in molecular exchanges between tumor cells and MSCs has been studied; however, some uncertainty remains regarding the nature and content of these EVs, as well as their effect on GBM progression. In this work we analyzed the effect of GBM EVs on the MSCs vesiculation profile. Surprisingly, we found out that GSCs MVs but not exosomes were taken up by MSCs, the consequence of which was an increased release of exosomes from these tumor-activated MSCs (TA-MSCs). The content of TA-MSC exosomes was also modified: we showed a significant decrease in EXO-miR-100-5p, EXO-miR-9-5p and EXO-let-7d-5p.

## 2. Materials and Methods

### 2.1. Materials

Unless stated otherwise, cell culture material was obtained from Thermo Fisher Scientific (Courtaboeuf, France) and chemicals from Sigma Aldrich (Lyon, France).

### 2.2. Patient Samples and Culture

Tumors were obtained from patients diagnosed with high-grade GBM from the “Tumorothéque IRCNA (Institut Régional du Cancer Nantes Atlantique)”. GBM primary cultures of GSCs and MSCs were obtained as described earlier [15,16,17]. GSCs were grown in defined medium (DMEM/HAM-F12, 2 mM L-glutamine, N2 and B27 supplement, 2 μg/mL heparin, 20 ng/mL EGF and 25 ng/mL bFGF, 100 U/mL penicillin and 100 μg/mL streptomycin). Bone marrow MSCs were cultured in MEMα containing ribonucleosides and deoxyribonucleosides supplemented with 10% fetal calf serum, 2 mM L-glutamine, 100 U/ml penicillin, and 100 μg/mL streptomycin. Cells were cultured in an incubator at 37 °C, 5% CO_2_ and 95% humidity.

### 2.3. Purification of Extracellular Vesicles

EVs were obtained as described earlier [18,19]. Briefly, after 48 h cell culture, supernatant was cleared of cells and cell debris by centrifuging for 10 min at 400× *g*. The resulting supernatant was then centrifuged 10 min at 2000× *g* to remove smaller cell debris, producing the conditioned media (CM). MV pellet was then purified from CM after centrifugation for 30 min at 10,000× *g* at 4 °C. The supernatant was saved for exosomes isolation, while the pellet was washed with phosphate buffered saline (PBS) and centrifuged again for 30 min at 10,000× *g* at 4 °C. The resulting MV pellet was then stored at −80 °C. The MV supernatant was ultracentrifuged in an Optima XE ultracentrifuge (Beckman Coulter) for 1 h at 100,000× *g* at 4 °C to pellet exosomes. Exosomes were washed with PBS and ultracentrifuged again for 1 h at 100,000× *g* at 4 °C, and finally stored at −80 °C.

### 2.4. Quantification of Cell Number and Protein Content

Cell viability was assessed by cell counting on Malassez chamber after Trypan blue staining. Protein concentration was determined using BCA protein assay (Thermo Fisher Scientific).

### 2.5. Nanosight Measurement of Particles Size and Concentration

Nanoparticle tracking analysis (NTA) was performed using the NS300 system (Nanosight, Malvern Panalytical, Palaiseau, France). Briefly, MV and exosome pellets were resuspended and diluted in filtered PBS to reach a concentration between 10^7^ and 10^9^ particles/mL. Particle suspensions were then injected into the system. The acquisition settings were chosen at the beginning of the measurement (Temperature 25 °C, Exposure 15, Gain 1) and maintained throughout the experiment. The algorithm analyzed the size distribution and concentration of EVs, based on 5 individual videos of 1 min per sample. Data were then processed in two different ways: either the size distribution of EVs was reported on a line graph, displaying particle concentration according to particle size (nm), or particles size/concentration were averaged for each sample to compare data between different conditions, and plotted as bar graphs. When indicated, particle concentration was normalized to the number of cells in the sample, taking into account the proliferation occurring during incubation.

### 2.6. Electron Microscopy

Negative staining electron microscopy was performed at the Microscopy Rennes imaging center platform (MRic TEM) (University of Rennes 1, Rennes, France). The EVs were deposited on glow-discharged electron microscope grids for 1 min and then negatively stained with 2% uranyl acetate for 10 seconds. The samples were observed using a 120 kV electron microscope (JEM 1400, Jeol) equipped with a CCD camera (model Orius, Gatan). Micrographs were acquired using the camera in binning mode 1.

### 2.7. PKH67 Staining and Analysis of Fluorescence by Flow Cytometry and Fluorescent Microscopy

GSCs EVs were labeled with PKH67 (green fluorescent cell membrane dye; Sigma Aldrich) as per manufacturer’s instructions. After incubation with stained EVs, MSC cells were analyzed using a BD Accuri C6 cytometer (BD Bioscience, Le Pont de Claix, France). Some cells were also fixed in 4% paraformaldehyde for 10 min, washed with PBS, mounted with Prolong antifade mounting media (Thermo Fisher Scientific), and observed under a Zeiss Axiovert 200-M inverted microscope.

### 2.8. miRNA Expression

RNA was reverse transcribed using a miScript II RT kit (Qiagen, Courtaboeuf, France) and analyzed by qPCR with the miScript miRNA PCR Arrays Human Cancer PathwayFinder (Qiagen) on the Rotor-Gene Q (Qiagen), according to the manufacturer’s instructions.

### 2.9. Statistical Analysis

Results were analyzed on Prism 9.0 software (GraphPad Software) and expressed as mean ± SD for indicated number of separate experiments. Paired Student’s *t* test was used for statistical analysis. A *p* value of <0.05 was considered significant. * *p* < 0.05; ** *p* < 0.01; *** *p* < 0.001; **** *p* < 0.0001.

## 3. Results

### 3.1. Characterization of GBM Extracellular Vesicles

**Figure 1:**
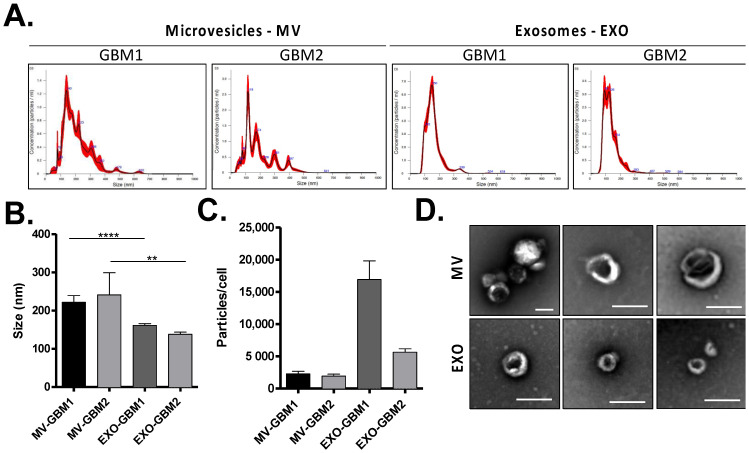
Characterization of GBM Extracellular vesicles. (**A**) Analysis of EV size distribution (microvesicles—MVs and exosomes—EXO) by NTA technology in GBM1 and GBM2 cell cultures. The mean size (**B**) and the concentration of particles per cell (**C**) were measured in both EV fractions. (**D**) Observation of GBM MVs and EXO by electron microscopy (bar scale = 200 nm). **: *p* value ≤ 0.01, ****: *p* value ≤ 0.0001.

To analyze the effect of GSC EVs on MSCs biology, we first purified two different populations of EVs by differential centrifugation: microvesicles (MV) and exosomes (EXO). To consider the variability between the different GBM subtypes, we chose to analyze EVs from 2 GSC primary cultures, where the size and concentration of GSC EVs were analyzed by NTA. Size measurements confirmed a difference in size between MVs and exosomes, with MVs having an average size superior to 200 nm and exosomes being smaller, with an average size of 150 nm (Figure 1A,B). The concentrations were also different between the two populations of EVs: after normalization to cell number, EV quantification showed that GSCs secreted more exosomes than MVs, especially in the GBM1 culture (Figure 1C). Observations of EVs by electron microscopy confirmed the difference in size between MVs and exosomes, and the presence of cup-shaped EVs (Figure 1D). 

### 3.2. Incorporation of GBM MVs by MSCs

**Figure 2:**
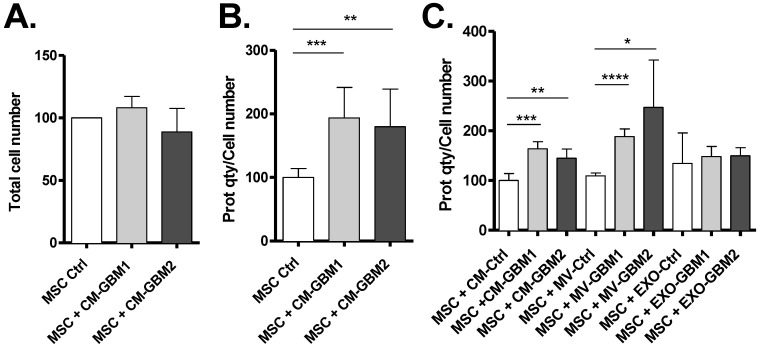
Exposure of MSCs to GSC Microvesicles (MVs) results in protein content increase. Incubation for 24 h of MSCs with the conditioned media (CM) from GBM1 or GBM2 primary cultures did not alter cell number (**A**) but increased their protein content (**B**). The differential transfer of MV or EXO fractions showed that the effect is mediated by MVs, suggesting their uptake by MSCs (**C**) (*n* = 3 independent experiments). *: *p* value ≤ 0.05, **: *p* value ≤ 0.01, ***: *p* value ≤ 0.001, ****: *p* value ≤ 0.0001.

The following step consisted of incubating conditioned media (CM) from GSC cultures with MSCs, to determine if some molecules or vesicles secreted by GSCs could influence MSCs. After 24 h incubation with GBM1 and GBM2 GSC CM, no change in MSC number was noticed (Figure 2A). However, quantification of protein content per cell indicated an increased protein concentration in these MSCs (Figure 2B), suggesting the incorporation by MSCs of molecules secreted into the CM of GSCs. In order to determine if GSC EVs participated in this process, similar experiments were performed using purified MVs and exosomes from CM of GSCs. As previously, no significant difference in MSC number was detected. However, the increase in protein quantity per cell observed after incubation of MSCs with GSC CM was similar to that observed after incubation with MVs alone (Figure 2C). Moreover, the addition of GSC exosomes did not modify MSC protein content. Overall, these results show that MVs secreted by GSCs influence protein content of MSCs, probably through their direct incorporation by these cells.

**Figure 3:**
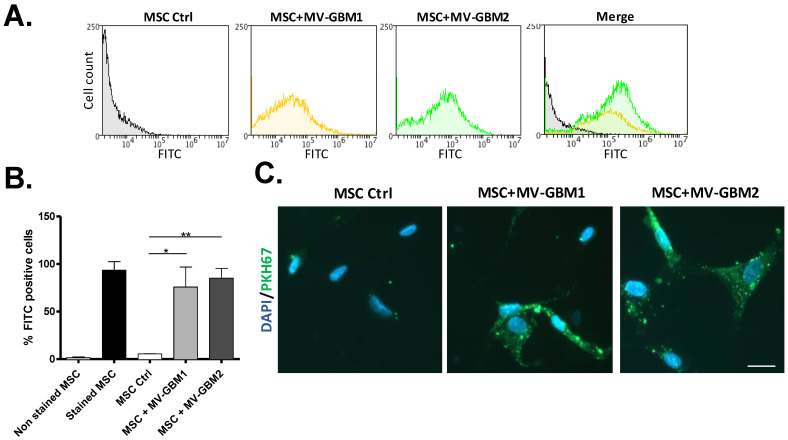
Uptake of GSC microvesicles by MSCs. (**A**,**B**) Flow cytometry analysis of MSCs incubated with PKH67-labelled MVs from either GBM1 or GBM2. FITC histograms are shown (**A**) as well as bar graph percentage of FITC positive cells (**B**). Corresponding pictures of fluorescent microscopy show fluorescence inside MSCs, suggesting the presence of PKH67-labelled MVs (**C**) (bar scale = 20 µm). *: *p* value ≤ 0.05, **: *p* value ≤ 0.01.

To confirm GSCs MVs uptake by MSCs, purified GBM1 and GBM2 MVs were stained with a cell membrane label, PKH67, before incubated for 24 h with MSCs where fluorescence gain was measured by flow cytometry. MSCs exposed to GBM1 and GBM2 MVs revealed a significant fluorescence increase compared to the control (Figure 3A), reaching close to 80% of MSCs positive for PKH67 staining (Figure 3B), validating the uptake of GSC MVs by MSCs. Fluorescence acquisition was also confirmed by fluorescent microscopy, showing the presence of fluorescent intracellular particles in MSCs incubated with GBM1 and GBM2 MVs (Figure 3C), and attesting the actual incorporation of MVs into MSC cells. No significant fluorescence intensity difference was detected in MSCs with GBM1 and GBM2 MVs.

### 3.3. MSCs Uptake of GBM MVs Alters Their Exosome Release

**Figure 4:**
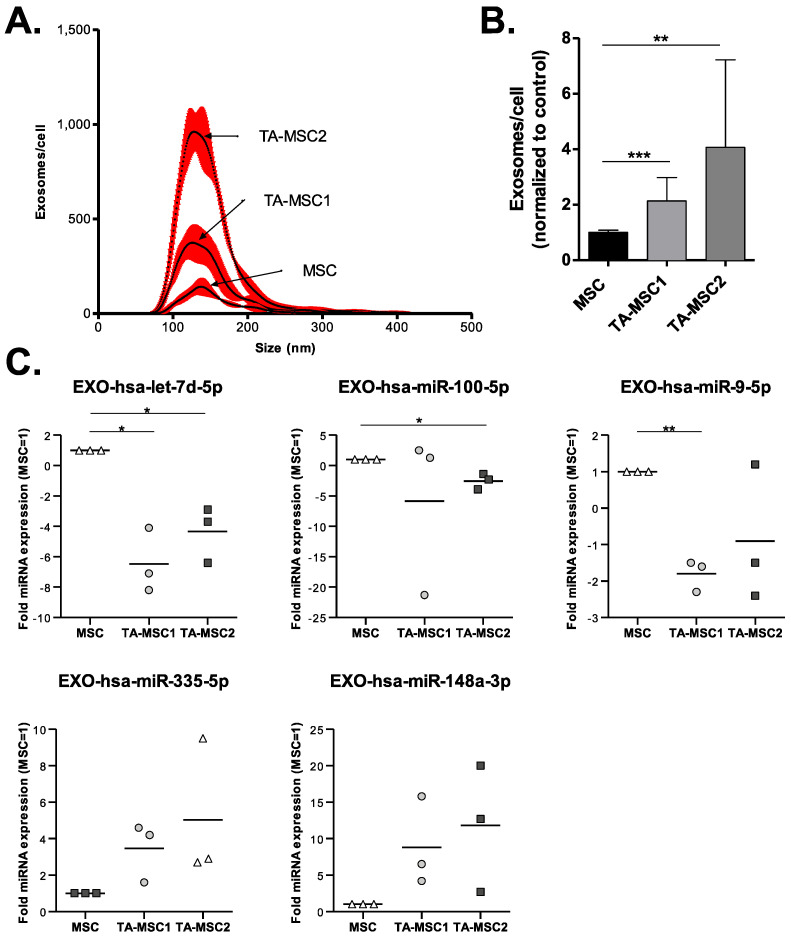
Exposure of MSCs to GBM microvesicles (MVs) leads to an increase in their exosome release and a modification of their exosome-miRNA profile. (**A**,**B**) NTA quantification of exosomes released by MSCs previously exposed to GBM1 and GBM2 MVs was performed and expressed as concentration of exosomes/cell depending on size (**A**), or the concentration of exosomes/cell normalized to control MSCs (**B**). (**C**) Expression of miRNAs present in exosomes of MSCs activated by GBM MVs, expressed as fold expression compared to control MSCs. (*n* = 3 independent experiments). *: *p* value ≤ 0.05, **: *p* value ≤ 0.01, ***: *p* value ≤ 0.001.

We then investigated whether activation of MSCs by GBM MVs (leading to Tumor-activated MSCs, TA-MSCs) could modify their vesiculation profile. NTA analysis of the exosome fraction, purified from the supernatant of MSCs exposed to GBM EVs, showed a significant increase in exosomes release (Figure 4A,B). Moreover, the miRNA content of the exosomes was also altered (Figure 4C and Supplementary Figure S1). In particular, we observed a significant decrease in exosomal let-7d-5p in MSCs activated by both GBM primary cultures; a similar profile was observed for miR-100-5p and miR-9-5p except the decrease was significant with only one of the 2 GSC primary cultures (Figure 4C–upper panel). In parallel we observed an increase in miR-335-5p and miR-148a-3p in exosomes purified from MSCs activated by both GSC MVs, albeit the difference was not significant (Figure 4C–lower panel). 

## 4. Discussion

The activation of stromal cells into cancer-associated fibroblasts (CAFs) by tumor cells is now a well-described process explaining how tumor can recruit and change the phenotype of stromal cells, including MSCs, to contribute to tumor growth and metastatic formation [20]. Signals triggering conversion of stromal cells into CAFs include amongst others transforming growth factor-β (TGFβ) family ligands, pro-inflammatory cytokines or direct cell-cell contact [21]. Consequently, those signals activate different pathways, including Janus kinase (JAK)–STAT signaling, SMAD and STAT transcription factors, or the contractile cytoskeleton. This results in a CAF phenotype, that can be defined by its elongated spindle morphology, absence of epithelial or endothelial markers, expression of mesenchymal markers (vimentin, α-smooth muscle actin (α-SMA), fibroblast activation protein (FAP), or platelet-derived growth factor alpha (PDGF-α)), and absence of mutations associated with cancer. Several studies showed that tumor EVs can also ensure this activation function [22,23,24]. For example, exosomes can generate CAFs through the transmission of growth factors involved in fibroblast activation, such as TGF-β from bladder cancer cells that activates SMAD pathway in fibroblasts [25], or the transfer of BMP from gastric cancer cells that activates PI3K/AKT and MEK/ERK pathways in pericytes [26,27]. Exosomes can also mediate CAF activation through the induction of endothelial-mesenchymal transition from melanoma cells [28], the transfer of miRNAs promoting β1-integrin-NF-κB signaling (from metastatic liver cancer cells) [29], cell motility and extracellular matrix remodeling pathways (breast cancer cells) [30] or SOCS1/JAK2/STAT3 signaling (melanoma cells) [31]. The exosomal transfer of cervical cancer Wnt2B activates Wnt/β-catenin signaling to activate fibroblasts into CAFs [32] while survivin from breast cancer induces CAFs through SOD1 upregulation [33]. The transfer of exosomes derived from colorectal cancer cells to fibroblasts also induces a dramatic change in their proteomic profile, correlating to phenotypes promoting proliferation, angiogenesis, invasion as well as metabolic reprogramming [34].

While several studies have already described the secretion of exosomes by tumor cells to generate CAFs, here we show that in GBM bigger EVs, such as MVs, can also be essential for the activation of stromal MSCs. GSCs MVs are bigger than exosomes but are secreted in lower quantity (Figure 1). Unexpectedly, when quantifying proteins in MSCs cells exposed to GSCs EVs, only MVs seemed to induce a change in protein content in recipient cells (Figure 2), showing MVs are actually internalized by MSCs and not exosomes (Figure 3). 

Few articles pointed out an involvement of MVs in CAF activation. However, this must be reinterpreted in the light of the progress made regarding the definition of EVs since the distinction between exosomes and MVs was not clearly made then. Two studies claimed the activation of stromal cells into CAFs by MVs [35,36], but EVs were actually prepared from a 100,000g ultracentrifugation fraction, which corresponds to a mix of MVs and exosomes. This confusion highlights the need for an universal EV classification and procedure definition, that the International Society for Extracellular Vesicles (ISEV) is actively working on for several years, with the edition of Minimal Information for Studies of Extracellular Vesicles (“MISEV”) guidelines [9]. 

Similarly, Antonyak et al. first described the activation of fibroblasts by breast cancer MVs [37], while the EV fraction was in fact purified after 100,000g ultracentrifugation (containing exosomes too). Interestingly though, more recently the same research team renewed the experiment with a filtration protocol excluding exosomes, showing that breast cancer MVs can indeed activate fibroblasts into CAFs, but only on stiff matrices [38]. MVs from prostate cancer cells were also shown to activate fibroblasts through ERK1/2 phosphorylation, and the resulting CAFs increased their secretion of MVs boosting cancer cell migration and invasion [39]. The extracellular matrix (ECM) was also described as an important factor, as matrix metalloproteinases (MMP) expression was increased inside tumor MVs as well as in CAFs. 

Overall, there are only two studies showing generation of CAFs after the uptake of MVs by stromal cells. Added to our work, it shows that the role of MVs in stromal cell activation into CAFs may have been underestimated, outshined by the success of exosomes. Further research would be needed to detail the modalities of this MV-mediated CAF activation, keeping in mind that the ECM plays a critical part in the TME and the establishment of the CSC niche [40,41] in GBM and many other tumor types. The development of new bioengineered 3D tumor models would become essential for further answers. We could actually wonder whether the differences between GSCs neurosphere culture compared to GBM adherent monolayer culture could explain the difference in CAF activation by MVs versus exosomes, in line with the involvement of ECM in the intake of MVs by stromal cells as suggested previously [38]. Astonishingly, culture of GBM cells in 3D ECM microenvironment correlates to metabolic changes [42], reflecting TME influence on GBM bioenergetics [43]. Likewise, we noted in one of our previous works a metabolic reprogramming in GSC/TA-MSC organoids, linked to the transfer of mitochondria through tunneling nanotubes and extracellular vesicles [16].

We also show that the activation of MSCs into TA-MSCs increases their production of exosomes (but not MVs) and modifies the miRNA profile of these exosomes (Figure 4), as observed in CAFs. It has now been described many times that modifications associated with the activation of stromal cells into CAFs include alterations in the vesiculation process, impacting the proliferation, migration or drug resistance in tumor cells [24,44]. In particular, a change in exosomal miRNAs seems to be a recurrent observation after CAF activation [24,45,46]. 

In particular, under our conditions we show that 3 miRNAs are significantly decreased in TA-MSCs, while 2 miRNAs are increased. MiR-100-5p downregulation in pancreatic ductal adenocarcinoma (PDAC) [47], oral squamous cell carcinoma [48], prostate cancer [49,50], lung cancer [51], bladder cancer [52], endometrial carcinoma [53] and breast cancer [54,55,56] contributes to tumor progression by regulating cell tumor proliferation, the response to therapy, migration, invasion or stemness. MiR-9 is also described as a negative regulator of tumor cell proliferation, invasion or drug resistance in pancreatic cancer cells [57], PDAC [47], hepatocellular carcinoma [58], gastric cancer [59], prostate cancer [60] or GBM [61,62,63]. The non-coding RNA let-7d can have different targets and effect in tumors [64]. Interestingly, let-7d blocks neural stem cell proliferation and promotes their neuronal differentiation and migration and its expression correlated to miR-9 expression [65]. Interestingly, the three miRNAs that are downregulated, miR-100-5p, miR-9-5p and let-7d-5p, were all shown to be involved in tumor suppression. They were also involved in IGFR-1 signaling [47], which promoted GBM survival [66]. A deeper analysis of regulators of this pathway would therefore be interesting.

In parallel, while the difference did not appear significant, we found interesting that miR-148a-3p and miR-335-5p were upregulated in TA-MSCs. MiR-148a-3p is a crucial regulator of GBM progression, by promoting tumor stem cell proliferation, migration, invasion and angiogenesis [67,68,69,70,71,72], while miR-335-5p has been shown to be upregulated in GBM [73,74]. 

While complement experiments will be needed to precise the effect of TA-MSCs exosomes on GBM cells, the change in miRNA we identified clearly suggest that they promote GBM progression.

## 5. Conclusions

Overall, our work emphasizes once again the critical role played by EVs in tumor progression, more specifically in the communication between GBM cells and the stromal cells present in the TME. Unexpectedly, we uncover the participation of MVs, and not exosomes, in the activation of MSCs into CAFs-like cells. On the contrary, resulting TA-MSCs increase their secretion of exosomes, in which we observed a decrease in anti-tumor miRNAs (miR-100-5p, miR-9-5p and let-7d-5p).

## Supplementary Materials

The following supporting information can be downloaded at: https://www.mdpi.com/article/10.3390/biomedicines10010112/s1, Figure S1: Expression of miRNAs present in exosomes of MSCs activated by GBM MVs, expressed as fold expression compared to control MSCs.

## Abbreviations

CAF	Cancer-associated fibroblast
CM	conditioned media
ECM	extracellular matrix
EV	extracellular vesicles
GBM	Glioblastoma
GSC	Glioblastoma stem cell
MSC	Mesenchymal Stem Cell
MV	microvesicle
NTA	Nanosight tracking analysis
PDAC	pancreatic ductal adenocarcinoma
TA-MSC	Tumor-Activated MSC
TME	tumor microenvironment.
