# Supplementary figures and images for: The Activation of Mesenchymal Stem Cells by Glioblastoma Microvesicles Alters Their Exosomal Secretion of miR-100-5p, miR-9-5p and let-7d-5p
Source: Biomedicines. 2022 Jan 6;10(1):112. doi: 10.3390/biomedicines10010112 (PMC8773192; doi:10.3390/biomedicines10010112)

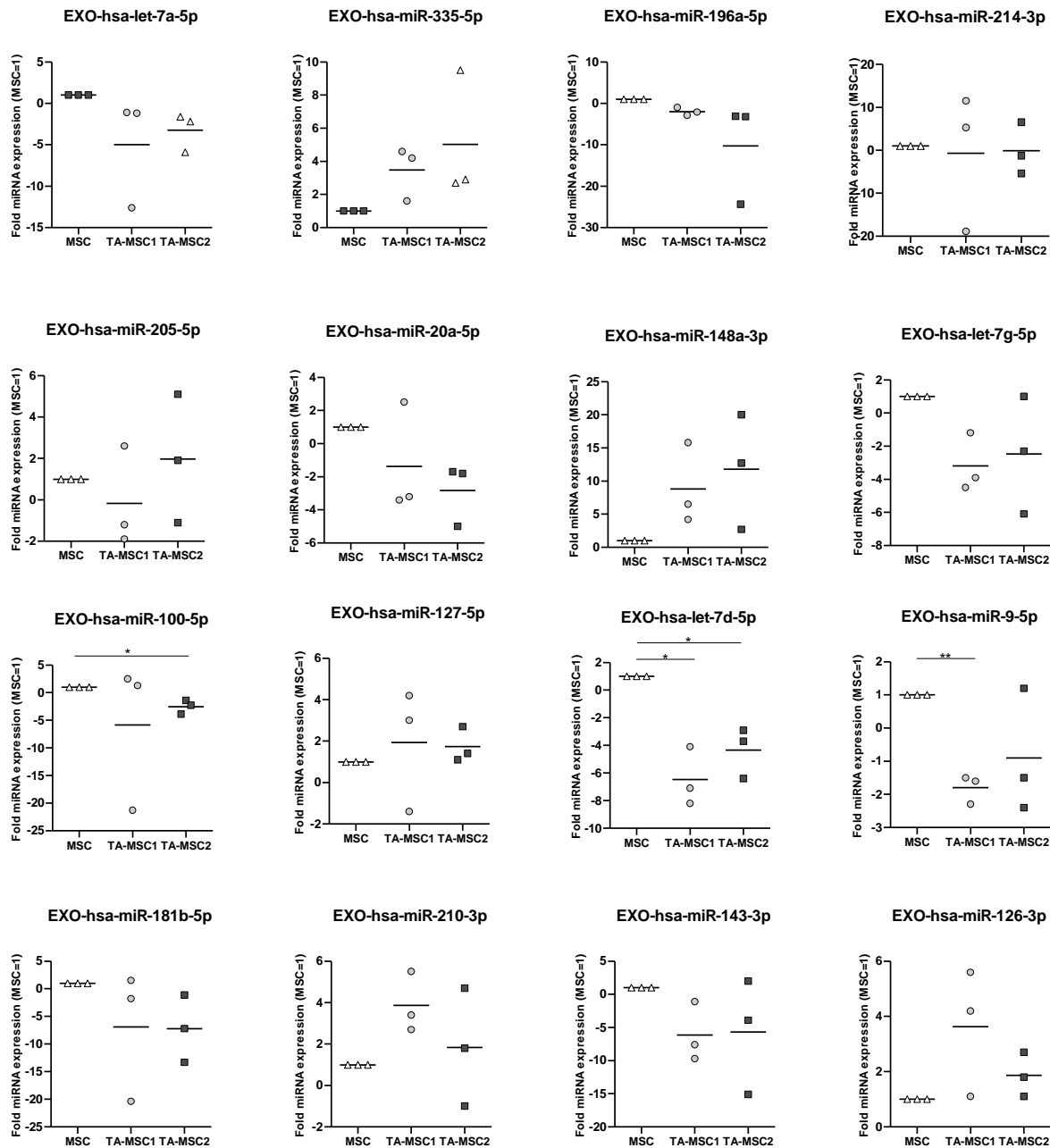

Supplementary Figure S1

Supplement: Supplementary file 1 [file biomedicines-10-00112-s001.zip › biomedicines-1510711-supplementary.pdf]
